# A novel one-step approach for the construction of yeast surface display Fab antibody libraries

**DOI:** 10.1186/s12934-017-0853-z

**Published:** 2018-01-09

**Authors:** Simon Rosowski, Stefan Becker, Lars Toleikis, Bernhard Valldorf, Julius Grzeschik, Deniz Demir, Iris Willenbücher, Ramona Gaa, Harald Kolmar, Stefan Zielonka, Simon Krah

**Affiliations:** 1 Institute for Organic Chemistry and Biochemistry, Technische Universität Darmstadt, Alarich-Weiss-Strasse 4, 64287 Darmstadt, Germany; 2 Protein Engineering and Antibody Technologies, Merck KGaA, Frankfurter Strasse 250, 64293 Darmstadt, Germany; 3 Chemical and Pharmaceutical Development, Merck KGaA, Frankfurter Straße 250, 64293 Darmstadt, Germany

**Keywords:** Golden Gate Cloning, Yeast surface display, Fab fragment, Antibody discovery

## Abstract

**Background:**

Yeast surface display (YSD) has proven to be a versatile platform technology for antibody discovery. However, the construction of antibody Fab libraries typically is a tedious three-step process that involves the generation of heavy chain as well as light chain display plasmids in different haploid yeast strains followed by yeast mating.

**Results:**

Within this study, we aimed at implementing a focused Golden Gate Cloning approach for the generation of YSD libraries. For this, antibodies heavy and light chains were encoded on one single plasmid. Fab display on yeast cells was either mediated by a two-directional promoter system (2dir) or by ribosomal skipping (bicis). The general applicability of this methodology was proven by the functional display of a therapeutic antibody. Subsequently, we constructed large antibody libraries with heavy chain diversities derived from CEACAM5 immunized animals in combination with a common light chain. Target-specific antibodies from both display systems were readily obtained after three rounds of fluorescence activated cell sorting. Isolated variants exhibited high affinities in the nanomolar and subnanomolar range as well as appropriate biophysical properties.

**Conclusion:**

We demonstrated that Golden Gate Cloning appears to be a valid tool for the generation of large yeast surface display antibody Fab libraries. This procedure simplifies the hit discovery process of antibodies from immune repertoires.

**Electronic supplementary material:**

The online version of this article (10.1186/s12934-017-0853-z) contains supplementary material, which is available to authorized users.

## Background

During recent decades, Yeast Surface Display (YSD) has proven to be a versatile technology for antibody engineering and hit discovery [1–7]. Pioneered by Boder and Wittrup in 1997, the genotype–phenotype coupling principle was initially demonstrated with an affinity maturation process of a FITC binding single chain antibody fragment (scFv) [1]. To this date, many other applications, for instance antibody stability- and pH-engineering [3, 8–10], antigen based selection of antibody repertoires from naïve [11] and synthetic sources [6, 12, 13] as well as from immunized animals were reported [2, 4]. Moreover, many non-conventional antibodies and scaffold proteins were engineered using this platform technology [6, 13–15]. Compared to other cell based selection systems like bacterial and phage display, one beneficial feature of YSD is the use of the eukaryotic expression host *S. cerevisiae* for the production of the displayed protein [1]. The presence of sophisticated quality control machineries residing in the endoplasmatic reticulum and Golgi apparatus might enable a more precise manufacturing of complex proteins in comparison to the prokaryotic host *E. coli* [1]. In addition, it’s compatibility with fluorescence activated cell sorting (FACS) enables real-time and on-line analysis as well as a fine discrimination of variants exhibiting different prescribed properties such as affinity or stability.

In the context of antibody discovery, the display of a variety of antibody formats are described in literature, ranging from simple antibody fragments like scFvs over Fab-fragments to full length IgGs [1, 4, 5, 16]. The classical approach for the display of e.g. Fab-fragments relies on the individual generation of heavy and light chain plasmids encoding regions VH-CH1 and VL-CL, respectively, via homologous recombination in haploid yeast strains. Afterwards, these haploid yeast cells can be combined into diploid cells that display functional Fabs on their surface by yeast mating [2, 5]. In the most common experimental setting, the surface display of the antibody variant is achieved by genetic fusion of the heavy chain segment to Aga2p, a cell surface-exposed protein that is anchored together with Aga1p in the yeast cell wall [1]. Upon co-expression of the light chain, assembly of the heterodimeric heavy and light chain fragment occurs leading to cell-surface exposed Fab [2, 5]. Although, this technology allows for the efficient generation of large antibody libraries, the multi-step process of library generation is tedious and time-consuming.

In 2008, a novel cloning technology was described, referred to as Golden Gate Cloning [17]. This cloning strategy has its origins in 1996, when it was shown that multiple DNA fragments can be cloned into a plasmid by the use of type IIs restrictions enzymes and T4 DNA ligase [18, 19]. Type IIs restriction enzymes are able to cleave outside of their recognition site, resulting in a DNA overhang which can be composed of any nucleotide sequence. Marillonnet et al. designed the cleavage sites and the resulting overhangs of two DNA fragments in a way that both digested fragments were ligated to a product in a seam-less manner [17]. This allowed for sub-cloning in a single step and a single tube with a cloning efficacy close to 100%. A major advantage of this cloning method is the independence of the enzyme recognition site from the gene of interest and that the recognition site can be designed to be eliminated during the restriction. In addition, the cleavage site overhangs can be composed of different distinct sequences (herein termed signature sequences) enabling directional cloning of several DNA fragments and preventing religation of the respective vectors [17]. Consequently, it was shown that up to ten different fragments can be assembled in a defined order by the generation of shuffling libraries, which can improve the outcome of library selection as in the case of trypsinogen variants displaying higher production titers compared to the wild-type protein [20].

**Fig. 1:**
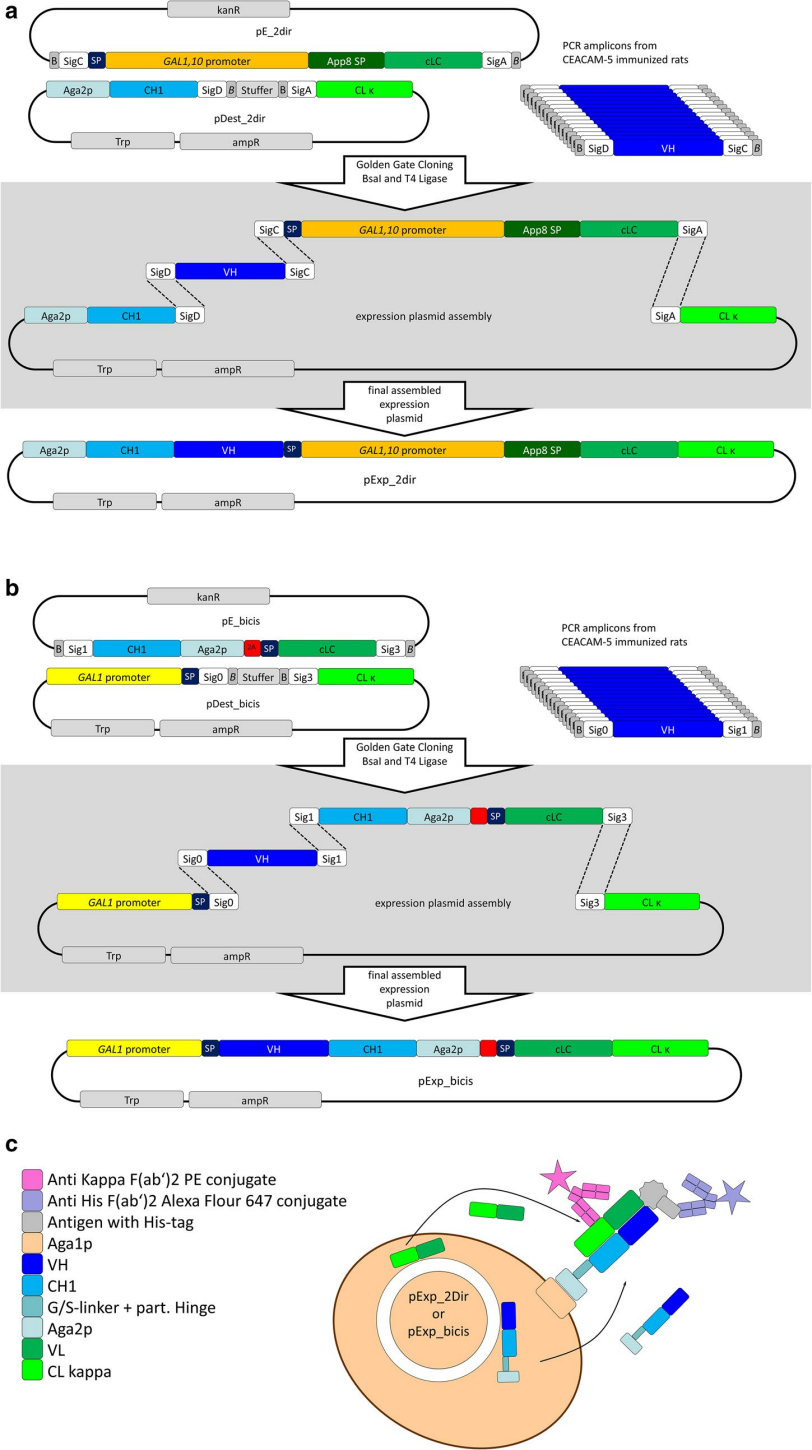
One step generation of YSD plasmids for the construction of large combinatorial Fab immune libraries using Golden Gate Cloning. Destination plasmids (pDest), entry plasmids (pE) and PCR amplicons contain or are flanked by *Bsa*I recognition sites in different orientations (B: ggtctcn, *B*: ngagacc). A linear and distinct assembly of those DNA fragments is ensured by the design of complementary signature sequences in defined order within the three modules after *Bsa*I cleavage. **a** The two-directional (2dir) display system enables the expression of the VH-CH1-Aga2p (Aga2p-signal-sequence; SP) gene product under control of the *GAL1*-promoter whereas the cLC-CLkappa (app8-signal-sequence; App8 SP) gene product is generated under control of the *Gal10*-promoter. **b** The bicistronic display system (bicis) allows for the expression of Fab-fragment heavy and light chains under control of the *GAL1*-promoter. The generation of distinct VH-CH1-Aga2p (Aga2p-signal-sequence; SP) and cLC-CLkappa (app8-signal-sequence; App8 SP) proteins is mediated by ribosomal skipping due to the T2A (2A) peptide. **c** Schematic illustration of Fab-fragments displayed on the surface of yeast cells. Genes are encoded by a single plasmid and expression is either conducted by two-directional promotors or by ribosomal skipping

In this work, we present a novel one-step Golden Gate Cloning approach for the generation of YSD Fab libraries enabling the simultaneous introduction of heavy chain and light chain variable regions into one single display vector. We demonstrate the versatility of this cloning technology for YSD by designing two different display strategies. In the two-directional system (2dir), expression of the heavy chain is enabled under the control of the *Gal1* promoter whereas light chain expression is facilitated via the *Gal10* promoter (Fig. 1a). In the bicistronic system (bicis), Fab display is mediated by ribosomal skipping (Fig. 1b) [21, 22]. We show that large antibody libraries with more than 10^8^ unique clones can readily be constructed by applying the herein presented approach. Furthermore, we prove that high-affinity antibodies can be isolated from such libraries using CEACAM5 immunized animals as a source of diversity. CEACAM5 [Carcinoembryonic antigen (CEA)] is a protein, expressed in most lung and breast cancer as well as gastrointestinal cells. Its overexpression is linked to liver metastasis, a main reason of death from colorectal cancer [23].

## Methods

### Immunization of transgenic rats

OmniRats^®^ [24–26], transgenic for human antibody variable regions, were immunized by genetic immunization using vaccination vectors encoding for CEACAM5 at Aldevron (Freiburg, Germany), as previously described elsewhere [2]. Experimental procedures and animal care were in accordance with EU animal welfare protection laws and regulations. We confirm that all experimental protocols were approved by a licensing committee from the local government (Landesuntersuchungsamt, Koblenz, Germany).

### Plasmids

Plasmids were designed in-house and synthesized at GeneArt (Thermo Fisher Scientific). Genetic elements were derived from pYD1 plasmid backbone (Yeast Display Vector Kit, version D, #V835-01, Thermo Fisher Scientific) as well as pESC vector series (Agilent). In general, entry plasmids (pEntry) were designed with a kanamycin resistance gene, whereas destination vectors contained an ampicillin resistance cassette as well as a tryptophan selection marker for selection in yeast (Fig. 1).

### Yeast strains and media

For antibody library construction, the *Saccharomyces cerevisiae* strain EBY100 (*MATa URA3*-*52 trp1 leu2Δ1 his3Δ200 pep4::HIS3 prb1Δ1.6R can1 GAL (pIU211:URA3*)) was employed (Thermo Fisher Scientific). Initially, EBY100 was cultivated in YPD medium composed of 20 g/L peptone, 20 g/L dextrose and 10 g/L yeast extract supplemented with 10 mL/L penicillin/streptomycin (Gibco). EBY100 cells harboring library plasmids (pDest) after Golden Gate Cloning were cultivated in medium using minimal SD-base (Clontech) with commercially available dropout mix (Clontech) composed of all essential amino acids except for tryptophan, according to the manufacturer’s instructions, supplemented with 5.4 g/L Na_2_HPO_4_ and 8.6 g/L NaH_2_PO_4_ × H_2_O. For induction of antibody gene expression, cells were transferred into respective SG dropout medium wherein glucose was replaced by galactose containing SG-base (Clontech). Additionally, 10% (w/v) polyethylene glycol 8000 (PEG 8000) was included [27, 28].

### Library construction

Total RNA was extracted from 1 × 10^7^ lymph node cells using RNeasy MiniKit (Qiagen) according to the manufacturer’s instructions. For cDNA synthesis 50 µL RNA extract, 20 µL RT-buffer, 40 µL 25 mM MgCl_2_, 20 µL 0.1 M DTT, 10 µL RNase Out and 10 µL Superscript III reverse transcriptase (SuperScript III First-Strand Kit, Thermo Fisher Scientific) was employed as well as random hexamer primers. Reaction conditions were: 5 min at 25 °C, 60 min at 50 °C followed by heat inactivation for 15 min at 85 °C. Afterwards, 1 µL RNase H was added followed by an incubation step at 37 °C for 20 min. Human antibody variable regions from OmniRats^®^ were amplified from cDNA in two successive PCR reactions using Q5 High-Fidelity 2 × Master Mix and 50 µL reaction volume (NEB). In PCR1, 12 different reactions were prepared with 5 µL cDNA using unique forward primers annealing to germline leader sequences and one reverse primer annealing to rat CH1 domain under the following conditions: 95 °C for 120 s, 30 cycles of 95 °C for 15 s, 58 °C for 30 s and 72 °C for 90 s. PCR products were purified via Wizard^®^ SV Gel and PCR Clean-up System (Promega). In the second PCR, human VH domains were amplified with primers incorporating *Bsa*I recognition sequences for subsequent Golden Gate Cloning. Primers according to Hust et al. [29] were modified and are given in Additional file 1: Table S1. In total for each library design (two-directional display as well as bicistronic display, Fig. 1), nine reactions were performed in parallel using forward primers 1S till 9S as well as reverse primer 9A. Reaction conditions were as follows: 98 °C for 30 s, 30 cycles of 98 °C for 10 s, 55 °C for 20 s and 72 °C for 30 s followed by 72 °C for 2 min. Afterwards, PCR products were purified via Wizard^®^ SV Gel and PCR Clean-up System (Promega). Finally, PCR products were pooled in an equimolar ratio. For verification of antibody display in both systems, the VH as well as VL of therapeutic antibody trastuzumab [30] were amplified utilizing primers HER2_VH_up and HER2_VH_lo as well as Her2_VL_up and Her2_VL_lo (Additional file 1: Table S1) using the same PCR conditions.

Construction of Fab display libraries was accomplished using Golden Gate Cloning. Reactions were performed in a final volume of 100 µL using 1 µg of respective destination vector, 2.2 µg of the respective entry vector (also encoding for light chain IGKV3-15*01, Fig. 1) and approx. 160 ng of pooled VH PCR product as well as 200 U *Bsa*I (New England Biolabs), 800 U T4 DNA ligase (New England Biolabs) and 10 µL 10× T4 Ligase buffer (New England Biolabs). Reaction conditions were 30 cycles of 1 min at 37 °C, 1 min at 16 °C followed by 5 min at 55 °C. After cloning, six reactions were pooled, purified using Wizard^®^ SV Gel and PCR Clean-up System (Promega) and eluted in a final volume of 30 µL which were subsequently used for one electroporation reaction into EBY100 as previously described by Benatuil et al. [31]. In general, five transformation reactions were performed for library establishment. Library sizes were calculated by plating out of serial dilutions. For proof of concept display of trastuzumab Fab, Golden Gate settings were slightly modified as follows: 1 µg of respective destination vector, 1.4 µg of the respective entry vector (Additional file 1: Figure S1), approx. 160 ng of VH PCR product and 160 ng VL PCR product as well as 200 U *Bsa*I (New England Biolabs), 800 U T4 DNA ligase (New England Biolabs) and 10 µL 10× T4 Ligase buffer (New England Biolabs).

### Library screening

Recombinant human His-tagged CEACAM5 extracellular domain as well as recombinant human His-tagged HER2 extracellular domain were purchased at R&D systems. For library screening, respective library cells were grown overnight in SD-Trp medium at 30 °C and 200 rpm. Afterwards, cells were transferred to SG-Trp medium at approximately 10^7^ cells/mL followed by incubation at 20 °C for 2 days. In general, antigen binding was detected by application of Penta-His Alexa Fluor 647 Conjugate antibody (Qiagen, 1:20 diluted in PBS). Fab display on the yeast surface was visualized using light chain specific goat F(ab’)_2_ anti-human kappa R-phycoerythrin (SouthernBiotech, 1:20 diluted in PBS). Labeling steps were performed with 10^7^ cells/20 µL on ice.

Labeling of cells for FACS analysis or sorting was conducted by two consecutive washing steps of library candidates with PBS followed by incubation with the respective antigen at a concentration of 1 µM. After incubation on ice for approx. 30 min an additional washing step was performed, followed by staining with Penta-His Alexa Fluor 647 Conjugate antibody as well as light chain specific goat F(ab’)_2_ anti-human kappa R-phycoerythrin. Finally, cells were washed with PBS. FACS-sorting rounds were either performed on a Sony SH800 cell sorter (Sony) or on a MoFlo Legacy cell sorter (Beckman Coulter).

### Sequencing of enriched yeast cell populations

Following sorting round three, plasmid DNA of yeast cells was isolated using 1 mL overnight culture and RPM^®^ Yeast Plasmid Isolation Kit (MP Biomedicals). Subsequently, obtained plasmids were used for transformation of electrocompetent *E. coli* Top10 cells (Invitrogen). Afterwards, 96 single clones were randomly chosen and grown overnight in 1 mL of LB-medium in a 96 deep well plate. The resulting cell suspensions were sent out for sequencing at Microsynth AG (Switzerland) with the following primers 2dir_seq_lo: CAGCAGTACCACCAGATGTAG and bicis_seq_lo: AACTGTTCATCAGATGGTGG.

### Expression and purification of isolated library candidates

VH regions as well as VL IGKV3-15*01 were cloned into pTT5 plasmids, which allow their expression as full-length IgG molecules in cell culture. Expi293 cells were transiently transfected with expression vectors following the instructions of the manufacturer (Thermo Fisher Scientific). Five days post transfection, antibody containing supernatants were harvested by centrifugation and purified by Antibody Purification Kit and Spin Columns with Prosep-A Media (Merck KGaA). After buffer exchange to PBS using Amicon Ultra-4 Centrifugal Filters (EMD Millipore) full length IgGs were analyzed by SDS-PAGE.

In addition aggregate formation was analyzed by analytical size exclusion chromatography. For this, a TSKgel SuperSW3000 column (4.6 × 300 mm, Tosoh Bioscience LLC) and an Agilent HPLC system was used. Differential scanning fluorometry on Prometheus NT.48 (Nanotemper Technologies) was applied to determine thermal stabilities of library candidates.

### Biolayer interferometry

Binding kinetic measurements were performed on the Octet RED96 system (ForteBio, Pall Life Science) at 30 °C and 1000 rpm agitation (ForteBio, Pall Life Science). Antibodies were loaded on anti-human Fc biosensors (AHC) at 5 µg/mL in PBS for 2 min. Afterwards, tips were transferred to kinetics buffer (KB; PBS, 0.1% Tween-20 and 1% bovine serum albumin, BSA) for 60 s for sensor rinsing. For kinetic analyses, association to CEACAM5 (varying concentrations ranging from 3.125 to 100 nM in KB) was measured for 400 s followed by dissociation for 900 s (in KB). In each experiment, one negative control was measured, where the captured antibody was incubated with KB instead of antigen. Data fitting and analysis was performed with ForteBio data analysis software 8.0 using a 1:1 binding model after Savitzky–Golay filtering.

## Results

### Design of library components

In order to investigate whether Golden Gate Cloning allows for library generation in yeast and subsequent isolation of antibodies starting from animal immunization, we decided to adopt a strategy for the isolation of common light chain antibodies which has been previously published by our group [2]. Heavy chain repertoires from immunized transgenic rats were combined with a single light chain. Although the probability of isolating high-affinity antibodies from such libraries is considered to be lower compared to approaches that focus on heavy- and light-chain repertoires after animal immunization, it was shown that this is a valid strategy to obtain so-called common light chain antibodies, which might be valuable for the generation of more complex antibody formats such as bispecific antibodies [32].

For library establishment as well as antibody display, two different expression strategies were developed. Both strategies have in common, that for screening of heavy chain diversities with a distinct light chain, three modules are utilized, a destination plasmid, an entry plasmid and the heavy chain variable region module i.e. the PCR amplified VH repertoire with flanking *Bsa*I sites and signature sequences allowing for cloning in a defined order (Fig. 1). The two-directional display system (Fig. 1a) is composed of an acceptor plasmid with a tryptophan marker allowing for selection in *S. cerevisiae.* Major components of this vector for antibody display are the CL-kappa region as well as the CH1 domain which is fused to Aga2p enabling display on the surface after Golden Gate Cloning (Fig. 1c). Between both components two *Bsa*I sites were introduced and separated by a short stuffer sequence. Signature sequences that allow for one-step cloning were part of CL-kappa (SigA) and CH1 (SigD). Signature sequences are given in Additional file 1: Table S2. The entry plasmid contains the *GAL1/10* promoters for expression of heavy and light chains, respectively, signal sequences for heavy and light chain secretion as well as a distinct VL (IGKV3-15*01) [2]. This entry module is flanked by signature sequences SigA as well as SigC of which the latter is incorporated into the signal peptide as well as *Bsa*I sites. PCR amplified VH repertoires from immunized transgenic rats are flanked with signature sequences (SigC and SigD) as well as *Bsa*I sites. All restriction sites were designed to be removed during digestion.

The bicistronic display system is composed of a single expression cassette under the control of the *GAL1* promoter (Fig. 1b). Simultaneous expression of heavy- and light-chains is enabled via the introduction of the picornaviral 2A peptide, mediating ribosomal skipping and therefore the lack of peptide bond formation between Aga2p which is fused to the heavy chain and the light chain. In contrast to internal ribosome entry sites, ribosomal skipping results in the translation of equal amounts of each protein and it has been shown previously that the 2A peptide is a versatile tool for yeast surface display [21, 33]. Similarly to the two-directional display system, signature sequences were constituents of sequence-encoding antibody constant regions or signal sequences and *Bsa*I sites were designed to be removed during restriction-ligation reaction, allowing for seamless one-step cloning of the final library display vector (pExp_bicis).

### Proof-of-concept: yeast surface display of a HER2-specific Fab

**Fig. 2:**
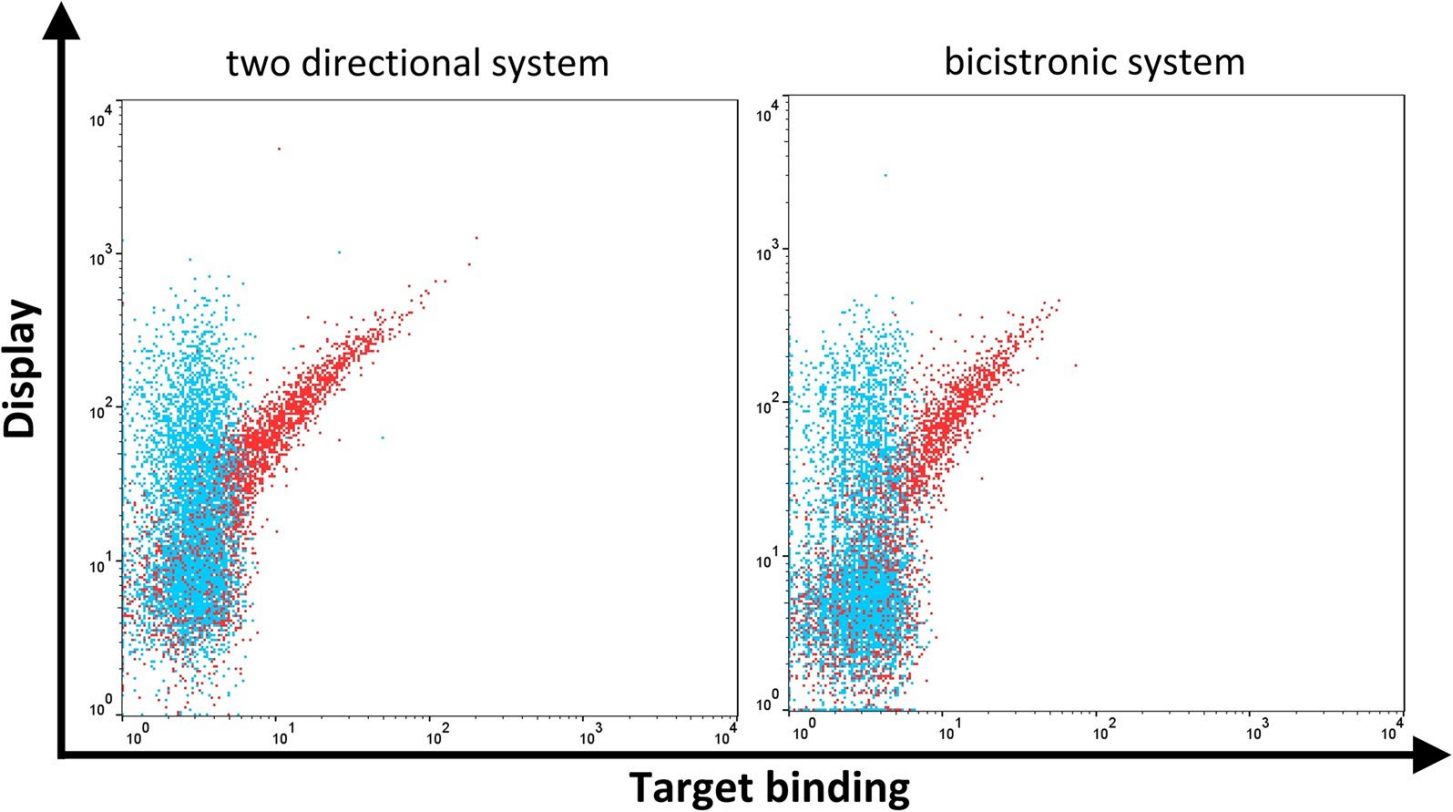
Overlay of trastuzumab displaying yeast cells either stained with detection antibodies only (blue) or with detection antibodies and HER2 as monitored by flow cytometry. Yeast cells were consecutively incubated with 1 µM of His tagged HER2 followed by secondary labeling with Alexa Fluor 647 conjugated anti-Penta-His antibody (target binding) and PE conjugated anti-kappa-antibody (display)

To validate whether this cloning strategy is valid for antibody YSD in general, we first aimed at displaying the HER2-specific Fab of the therapeutic antibody trastuzumab [30] on the yeast surface using both different vector strategies. A slightly modified cloning scheme was applied, allowing also for the introduction of light chains (Additional file 1: Figure S1). Consequently, entry plasmids were modified in a way that new signature sequences were inserted into the signal peptide mediating light chain expression (SigB for the two-directional system and Sig2 for the bicistronic system). Another entry module, the PCR-amplified VL of trastuzumab was also introduced and flanked with corresponding signature sequences and *Bsa*I recognition sites. Accordingly, VH and VL regions were amplified using primer sets as given in Additional file 1: Table S1. After Golden Gate Cloning, yeast cells were transformed according to Benatuil et al. [31]. Surface presentation of the trastuzumab Fab constructs from single clones of both display systems was characterized by indirect fluorescence labeling of the light chain constant region (CL-kappa) and binding of hexahistidine-tagged HER2 was detected using a fluorescently-labeled Penta-His-specific antibody. As shown in Fig. 2, single clones from both display strategies showed Fab surface expression as well as HER2-binding, clearly demonstrating the functionality of both display systems.

### Library construction and selection of CEACAM5-specific antibodies

We recently showed that potent CEACAM5-binding Fabs, encompassing a common light chain can be isolated via combination of animal immunization and yeast surface display screening [2]. For library generation via Golden Gate Cloning we applied the same animal-derived lymph nodes for cDNA generation, PCR amplification and eventually for library generation via Golden Gate Cloning. Following cDNA synthesis, heavy chain variable region repertoires were amplified in two consecutive PCR steps. Construction of final display vectors (pExp_2dir, pExp_bicis) was mediated using Golden Gate Cloning. Afterwards, six reactions were pooled and transformed into yeast cells as described by Benatuil et al. [31]. In total, five transformations were performed for each approach resulting in calculated library sizes of 1.3 × 10^8^ (2dir) and 6.9 × 10^8^ (bicis) unique clones, respectively. Sequencing of approximately 100 single clones from each library revealed a high correctness of 95% (2dir) and 93% (bicis), i.e. clones that contain functional Fab fragments without frame-shifts or stop-codons. Additionally, no sequence duplets were found in both approaches, indicating high heavy chain diversities of generated libraries.

**Fig. 3:**
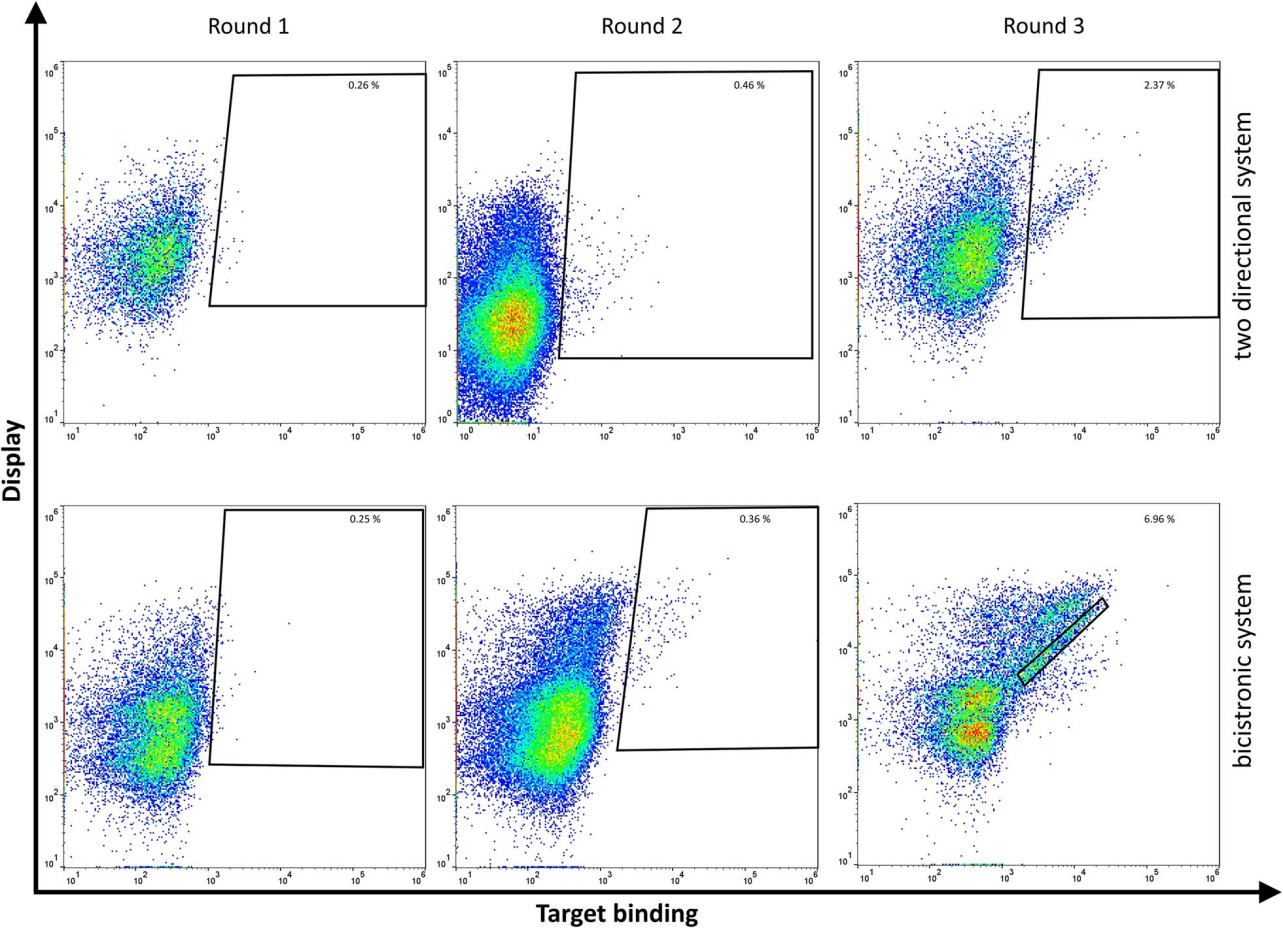
Screening of CEACAM5 common light chain YSD immune libraries by FACS. Yeast cells were consecutively incubated with 1 µM of His tagged CEACAM5 followed by secondary labeling with Alexa Fluor 647 conjugated anti-Penta-His antibody (target binding) and PE conjugated anti-kappa-antibody (display). Top: two-directional system; bottom: bicistronic system. In sorting round 3 (bicistronic system) the sorting gate was adjusted according to Additional file 1: Figure S2, since secondary reagent binders were detected

The two resulting libraries were screened separately by FACS for binders of recombinant human CEACAM5 protein. Target binding was identified by indirect antigen (His-tagged) fluorescence staining with Alexa Fluor 647 conjugated anti-Penta-His antibody. Fab-display levels were analyzed simultaneously by application of kappa light chain specific goat F(ab’)_2_ R-PE conjugate. Additionally, controls were introduced, in which respective cells were stained with detection antibodies only (data not shown). These controls were applied to adjust gates in a way that only antigen positive cell populations were considered within the gating strategy. During the first sorting rounds 0.25% (bicis) and 0.26% (2dir) double positive events were detected (Fig. 3) and a total of 1.9 × 10^8^ and 2 × 10^8^ cells were sorted for each of the different library approaches. Therefore, the theoretical maximum diversity of 1 × 10^7^ variants (lymphocytes used for RNA-extraction) was covered approximately 20-fold. During sorting rounds two and three minimally tenfold output of the previous sorting round was processed (Additional file 1: Table S3). While only a slight enrichment of antigen binding cells was observed in round 2 compared to the first sorting rounds (0.1–0.2%), the final FACS-plots (round 3) resulted in 2.4 and 7% double positives. Interestingly, in the bicistronic approach a significant fraction of cells showed secondary reagent binding against the Alexa Fluor 647 conjugated anti-Penta-His antibody (Fig. 3, Additional file 1: Figure S2). To avoid the isolation of such yeast cells, the sorting gate was adjusted accordingly and only cells were sorted that showed specific binding to CEACAM5.

**Fig. 4:**
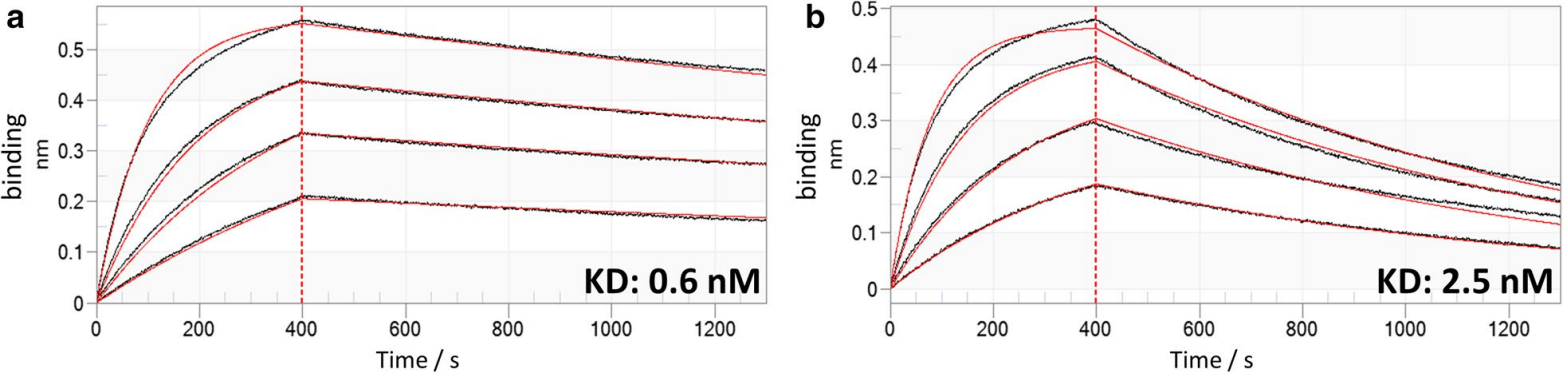
BLI sensorgrams of kinetic analyses of CEACAM5 binding to immobilized common light chain IgG variants 4G07 (**a**) and 4F02 (**b**). Association with recombinant CEACAM5 at concentrations of 25, 12.5, 6.25 and 3.125 nM was measured for 400 s while dissociation was measured for 900 s. Fitting (red lines) of binding curves (colored lines) was calculated using a 1:1 binding model and Savitzky-Golay filtering

A final analysis of both approaches after sorting round three demonstrated a distinct population of CEACAM5 binders with a strong correlation between Fab-display and antigen binding (Additional file 1: Figure S3). Finally, 100 single clones from both approaches were sent out for sequencing which revealed that the most frequently emerging VH sequences were the same in both libraries after sorting (Additional file 1: Figure S4). Interestingly, the two most abundant sequences showing less than 90% CDR sequence identity (Additional file 1: Figure S4) were also found to be the most prominent clones using the conventional library construction method of separate heavy and light chain vector construction followed by yeast mating [2]. These clones were afterwards reformatted into plasmids which enabled their expression as full length IgG molecules in Expi293 cells. After production and purification via Protein A spin columns, protein purity was analyzed by SEC and thermal stabilities were measured by differential scanning fluorimetry (Additional file 1: Figure S5). Less than 6% aggregates were determined and thermal stabilities were in the range of about 70 °C indicating favorable biophysical attributes of isolated library candidates. Moreover, binding kinetics to recombinant CEACAM5 protein were investigated via BLI. Specific high affinity antigen binding in the sub- and single-digit nanomolar range were found for clones from both libraries (Fig. 4), showing clear evidence that both approaches allow for the isolation of antigen-specific antibodies.

## Discussion

Yeast Surface Display has proven to be a versatile technology for antibody engineering and hit discovery [3, 5, 6, 11–13, 16]. Antibodies with favorable properties have been isolated from synthetic and naïve repertoires as well as from immunized animals [2, 4, 5, 11]. Along with these different sources of diversity, several antibody-formats can be displayed on the surface of yeast cells. While scFv based constructs can be easily expressed from single plasmids [4], a common technique for antibody Fab-display relies on the generation of heavy and light chains plasmids in haploid yeast strains, respectively [2, 5]. These can be combined by a process referred to as yeast-mating. As a result, the diploid yeast cells display functional Fab fragments on their surface. However, this three-step process of library generation is a sophisticated and laborious procedure.

Taking this into consideration, the present work describes a simplified one-step procedure for the generation of such diversities using Golden Gate Cloning. To demonstrate applicability of this novel system and to ensure comparability, lymph nodes from the same animal were utilized as starting material for library construction as our group already used for the generation of common light chain antibodies [2]. In our previous work, we demonstrated that common light chain antibodies can be isolated from immunized animals using YSD. Libraries were generated using the conventional three-step procedure resulting in a final library size of 2 × 10^8^ individual clones. Interestingly, generation of libraries using Golden Gate Cloning yielded in similar library sizes in a simplified process. Unlike the three-step approach, including construction of antibody chain pools by gap-repair driven homologous recombination and yeast mating that typically takes 2 weeks, the one-step Golden Gate Cloning procedure allows for the introduction of antibody diversities into yeast cells in only 1 week. Furthermore, sequencing of initially established libraries revealed a high diversity of heavy chain variants as well as a high proportion of functional clones, indicating that YSD libraries with suitable qualities can be generated using both Golden Gate Cloning approaches. Three rounds of sorting resulted in a significant enrichment of target binding populations from both libraries (Additional file 1: Figure S3). Interestingly, sequencing of enriched cell pools revealed identical antibody variants as already isolated by Krah et al. (Additional file 1: Figure S4), clearly demonstrating adequate functionality and quality of both display systems in combination with Golden Gate Cloning [2]. However, in the bicistronic display system a significant fraction of cells bound to the AlexaFluor647 conjugated anti-penta-His secondary detection antibody. This might be explained by the fact that antibody diversities derived from Omnirat immunizations were not naturally paired with the utilized common light chain. This may result in novel paratopes showing unspecific binding.

Since the advent of Golden Gate Cloning, as pioneered by Marillonnet et al. in 2008, a plethora of different constructs were genetically engineered [17, 20, 34, 35]. Related to this, this cloning technology was utilized for pathway manipulation in the yeast *Yarrowia lipolytica* [36]. The Golden Gate Cloning procedure has already been applied for the construction of plasmids used for complex immunological systems, such as the production of T cell receptor retroviral plasmids for gene transfer into primary T cells [37]. Additionally, this method has also been used for shuffling libraries for the isolation of protein variants with desired properties [20]. This study demonstrates that Golden Gate Cloning can also be successfully applied to antibody engineering and antibody discovery using YSD.

Within this study, we also aimed at designing and comparing two different display strategies in order to investigate whether one of both systems seems to be better suited for Fab antibody surface display. In the two-directional system, the *GAL1*/*GAL10* promoter is utilized for the expression of the heavy chain and light chain, respectively. Typically, the expression of proteins fused to this promoter system is induced more than 1000-fold by galactose [38, 39]. Even though it was shown that induction of proteins under the control of the *GAL1* promoter result in two- to fourfold higher activity [39], we were able to demonstrate that expression of the heavy chain Fab under the control of the *GAL1* promoter and expression of the light chain mediated by the *GAL10* promoter result in adequate antibody display levels (Figs. 2, 3).

In the bicistronic display system, a single expression cassette is finally produced under the control of the *GAL1* promoter. Equal expression of the heavy chain and the light chain is ensured by the use of the T2A peptide derived from *Thosea asigna* virus, mediating ribosomal skipping [40]. Since the 2A release site is located after the last glycine residue of the peptide sequence, the majority of the peptide is attached to the first protein. We therefore designed the expression cassette in a way that the residual peptide is attached to the C-terminus of Aga2p, since it is known that proteins can be fused at its C- and N-terminus without perturbing the functional integrity of Aga2p [2, 6]. Similarly, the last proline residue of the 2A peptide is the first residue of the second protein that is being translated, which was either the Aga2p signal peptide in the bicistronic library approach or the App8 signal peptide for the bicistronic display of HER2-targeting trastuzumab [41]. Consequently, this proline residue is not part of the finally processed and secreted light chain. Additionally, we were able to show that display levels were quite similar between both display systems, indicating that the proline residue does not negatively impact processing of both signal peptides. Ultimately, both display systems allowed for the generation of large antibody Fab libraries with similar unique clone numbers. Likewise also the isolation of identical target-specific antibodies proves that both approaches seem to be applicable YSD systems for antibody discovery and engineering. As both technologies allow the incorporation of antibody diversities into destination plasmids in a single step and identical binders were derived from both selection campaigns, a recommendation for either one of the display technologies based on the presented data cannot be given.

In this work, the light chain sequence was kept constant throughout the cloning and screening procedure since we aimed at isolating common light chain antibodies, where in theory antigen binding is mainly or exclusively mediated by the heavy chain [2]. Since large library sizes exceeding 10^8^ variants can easily be obtained, the Golden Gate Cloning strategies presented here may also be amenable to the simultaneous generation and combination of VH and VL repertoires. Notably, this could significantly simplify the isolation of conventional antibodies from immunized animals.

## Conclusion

The generation of antibody Fab immune libraries for YSD is a tedious and time consuming multistep process which includes the generation of heavy and light chain diversities in haploid yeast cells that need to be combined via yeast mating. In this work we describe a simplified procedure for the generation of such libraries based on Golden Gate Cloning. By application of this technology, combinatorial libraries can be easily constructed in just one single step.

The feasibility of the presented approach was demonstrated in a proof of concept study, in which large Fab repertoires were generated and high affinity common light chain antibodies were identified after three rounds of FACS utilizing two different antibody display approaches. According to our findings, this process might also be applicable for the random generation and combination of VH and VL diversities, which would significantly improve the antibody hit discovery process from immunized rodents.

## Additional file

**Additional file 1.** Additional tables and figures.

## Abbreviations

Bicis: bicistronic display system
BLI: biolayer interferometry
CDR: complementarity determining region
pDest: destination plasmid
DSF: differential scanning fluorimetry
pE: entry plasmid
pExp: expression plasmid
FACS: fluorescence activated cell sorting
2dir: two-directional display system
SEC: size exclusion chromatography
VH: variable domain of the heavy chain
VL: variable domain of the light chain
YSD: yeast surface display
