# Supplementary material for: A novel one-step approach for the construction of yeast surface display Fab antibody libraries
Source: Microb Cell Fact. 2018 Jan 9;17:3. doi: 10.1186/s12934-017-0853-z (PMC5759264; doi:10.1186/s12934-017-0853-z)
Supplement: Supplementary file 1 — Additional file 1. Additional tables and figures. [file 12934_2017_853_MOESM1_ESM.docx]

**A novel one-step approach for the construction of yeast surface display Fab antibody libraries**

Simon Rosowski^1^, Stefan Becker^2^, Lars Toleikis^2^, Bernhard Valldorf^3^, Julius Grzeschik^1^, Deniz Demir^2^, Iris Willenbuecher^2^, Ramona Gaa^2^, Harald Kolmar^1^, Stefan Zielonka^2^* and Simon Krah^2^*

^1^Institute for Organic Chemistry and Biochemistry, Technische Universität Darmstadt, Alarich-Weiss-Strasse 4, D-64287 Darmstadt, Germany

^2^Protein Engineering and Antibody Technologies, Merck KGaA, Frankfurter Strasse 250, D-64293 Darmstadt, Germany

^3^Chemical and Pharmaceutical Development, Merck KGaA, Frankfurter Straße 250, D-64293 Darmstadt, Germany

^*^To whom correspondence should be addressed: Stefan Zielonka (Stefan.Zielonka@merckgroup.com) and Simon Krah (Simon.Krah@external.merckgroup.com)

**Table S1:** Oligonucleotide primers

| Name | Sequence (5′–3′) |
| --- | --- |
| 1S_YSD_2dir | \| GCGCGCGCGGTCTCTAGGTGAGGTBCAGCTGGTGCAGTCTGG \| \| --- \| |
| 2S_YSD_2dir | GCGCGCGCGGTCTCTAGGTGARRTSCAGCTGGTRCARTCTGG |
| 3S_YSD_2dir | GCGCGCGCGGTCTCTAGGTGAGRTCACCTTGAAGGAGTCTGG |
| 4S_YSD_2dir | GCGCGCGCGGTCTCTAGGTGARGTGCAGCTGGTGGAGTCTGG |
| 5S_YSD_2dir | GCGCGCGCGGTCTCTAGGTGAGGTGCAGCTGKTGGAGWCYSG |
| 6S_YSD_2dir | GCGCGCGCGGTCTCTAGGTGAGGTGCARCTGCAGGAGTCGGG |
| 7S_YSD_2dir | GCGCGCGCGGTCTCTAGGTGAGSTGCAGCTRCAGSAGTSSGG |
| 8S_YSD_2dir | GCGCGCGCGGTCTCTAGGTGARGTGCAGCTGGTGCAGTCTGG |
| 9S_YSD_2dir | GCGCGCGCGGTCTCTAGGTGAGGTACAGCTGCAGCAGTCAGG |
| 9A_YSD_2dir | GCGCGCTGGTCTCTTAGTAGAAGCTGARGAGACRGTGACC |
| 1S_YSD_bicis | GCGCGCGCGGTCTCAAGGTGAGGTBCAGCTGGTGCAGTCTGG |
| 2S_YSD_bicis | GCGCGCGCGGTCTCAAGGTGARRTSCAGCTGGTRCARTCTGG |
| 3S_YSD_bicis | GCGCGCGCGGTCTCAAGGTGAGRTCACCTTGAAGGAGTCTGG |
| 4S_YSD_bicis | GCGCGCGCGGTCTCAAGGTGARGTGCAGCTGGTGGAGTCTGG |
| 5S_YSD_bicis | GCGCGCGCGGTCTCAAGGTGAGGTGCAGCTGKTGGAGWCYSG |
| 6S_YSD_bicis | GCGCGCGCGGTCTCAAGGTGAGGTGCARCTGCAGGAGTCGGG |
| 7S_YSD_bicis | GCGCGCGCGGTCTCAAGGTGAGSTGCAGCTRCAGSAGTSSGG |
| 8S_YSD_bicis | GCGCGCGCGGTCTCAAGGTGARGTGCAGCTGGTGCAGTCTGG |
| 9S_YSD_bicis | GCGCGCGCGGTCTCAAGGTGAGGTACAGCTGCAGCAGTCAGG |
| 9A_YSD_bicis | GCGCGCGCGCGCGCGGTCTCGAAGCTGARGAGACRGTGACC |
| HER2_VH_up_2dir | GCGCGCGGTCTCTAGGTGAGGTGCAGCTGGTTGAATC |
| HER2_VH_lo_2dir | GCGCGCTGGTCTCTTAGTAGAAGCGCTAGACACTGTGACCAG |
| HER2_VL_lo_2dir | GCGCGCGGTCTCTTTCTCTTGATTTCCACCTTGGTG |
| HER2_VL_up_2dir | GCGCGCTGGTCTCTAAGCGAGACATCCAGATGACACAG |
| Her2_VH_up_bicis | GCGCGCGGTCTCAAGGTGAGGTGCAGCTGGTTGAATC |
| Her2_VH_lo_bicis | GCGCGCGGTCTCGAAGCGCTAGACACTGTGACCAGGGT |
| Her2_VL_up_bicis | GCGCGCGGTCTCTAAGCGTGACATCCAGATGACACAG |
| Her2_VL_lo_bicis | GCGCGCGGTCTCTTTCTCTTGATTTCCACCTTGGTG |

**Table S2:** Signature Sequences. Sequences are given for specific signatures of two-directional display and bicistronic display. For common light chain display, plasmids were used instead of PCR products (see Fig. 1). Consequently, signature sequences SigB and Sig2 are not part of library design.

| **2dir** | |
| --- | --- |
| **Name** | **Sequence** |
| SigA | TTCT |
| SigB | AAGC |
| SigC | AGGT |
| SigD | TAGT |
|  | |
| **bicis** | |
| **Name** | **Sequence** |
| Sig0 | AGGT |
| Sig1 | AAGC |
| Sig2 | AAGC |
| Sig3 | TTCT |

**Table S3:** Statistical evaluation of analyzed and sorted cells during each sorting round of two directional and bicistronic CEACAM5 immune libraries.

|  | **two directional system** | | | **bicistronic system** | | |
| --- | --- | --- | --- | --- | --- | --- |
| **Sorting round** | **1** | **2** | **3** | **1** | **2** | **3** |
| **Cells analyzed in total** | 1.96 x 10^8^ | 0.94 x 10^8^ | 1.5 x 10^7^ | 2 x 10^8^ | 5,78 x 10^7^ | 7.5 x 10^5^ |
| **Sorted cells** | 8.9 x 10^5^ | 3.3 x 10^5^ | 1.6 x 10^5^ | 5 x 10^5^ | 1,7 x 10^5^ | 2.5 x 10^4^ |

**
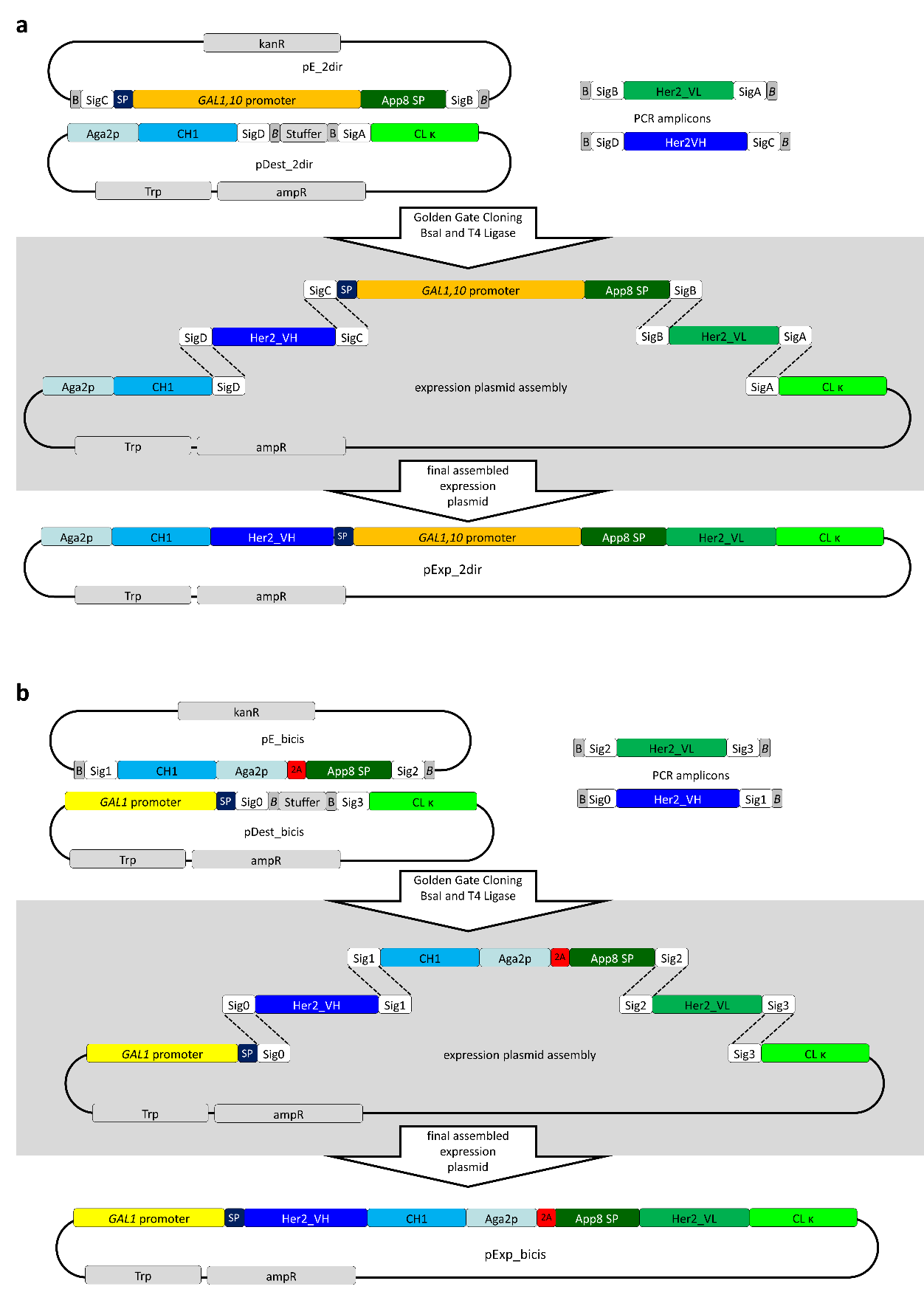
**
**Figure S1:** One step generation of YSD plasmids for the construction of trastuzumab Fab single clones using Golden Gate Cloning. Destination plasmids (pDest), entry plasmids (pE) and PCR amplicons contain or are flanked by *Bsa*I recognition sites in different orientations (B: ggtctcn, *B*: ngagacc). A linear and distinct assembly of those DNA fragments is ensured by the design of complementary signature sequences in defined order within the four modules after *Bsa*I cleavage. **(a)** The two-directional (2dir) display system enables the expression of the VH-CH1-Aga2p (Aga2p-signal-sequence; SP) gene product under control of the *GAL1*-promoter whereas the cLC-CLκ (app8-signal-sequence; App8 SP) gene product is generated under control of the *GAL10*-promoter. (**B**) The bicistronic display system (bicis) allows for the expression of Fab-fragment heavy and light chains under control of the *GAL1*-promoter. The generation of distinct VH-CH1-Aga2p (Aga2p-signal-sequence; SP) and cLC-CLκ (app8-signal-sequence; App8 SP) proteins is mediated by ribosomal skipping due to the T2A (2A) peptide.


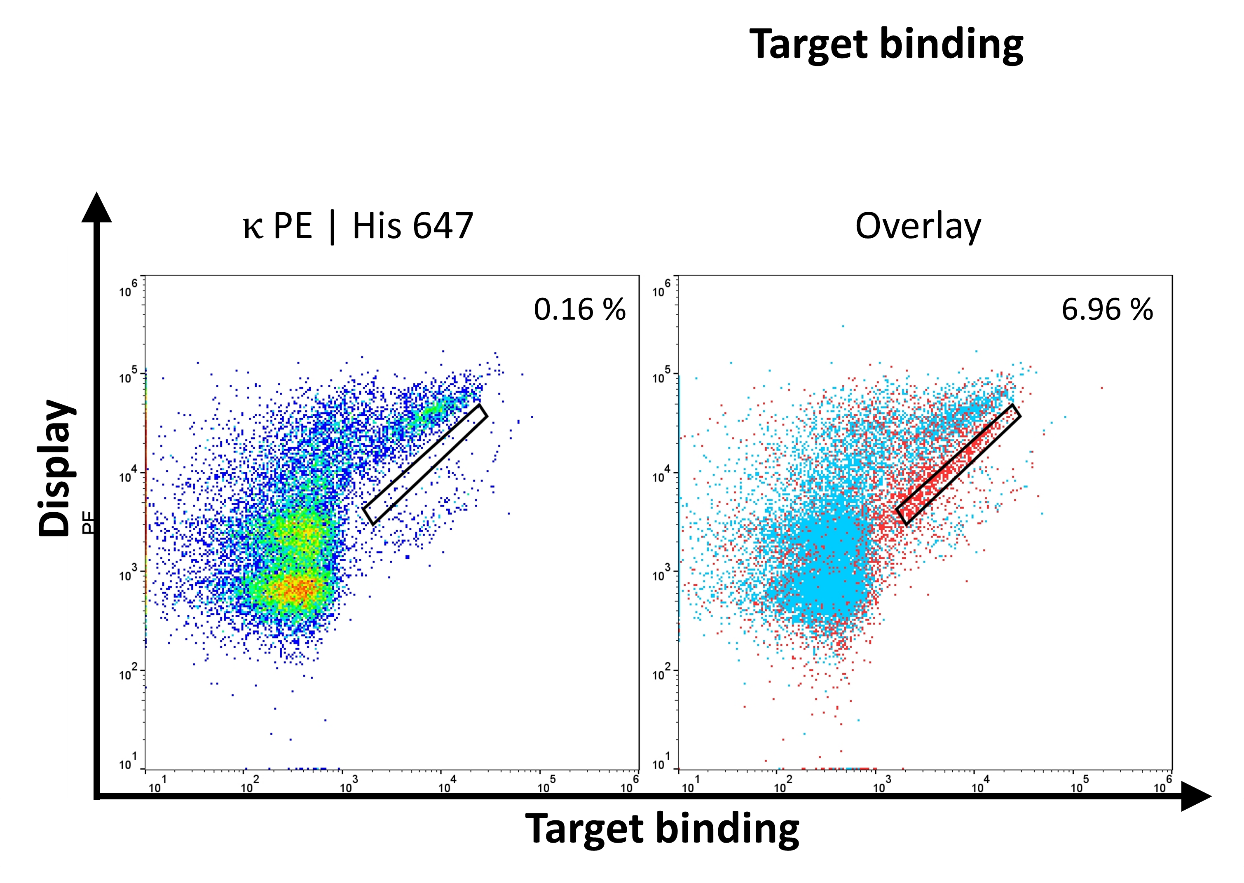


**Figure S2:** Target binding analysis of the bicistronic CEACAM5 display system after two rounds of sorting monitored by FACS. Cells were either stained with PE conjugated anti-kappa-antibody, anti-HIS-647 antibody and CEACAM5 or with detection antibodies only. Left: Cells were stained with detection antibodies only. Right: Overlay of cells stained with detection antibodies only (blue) and cells stained with detection antibodies and CEACAM5 (red). Sorting gates are indicated.


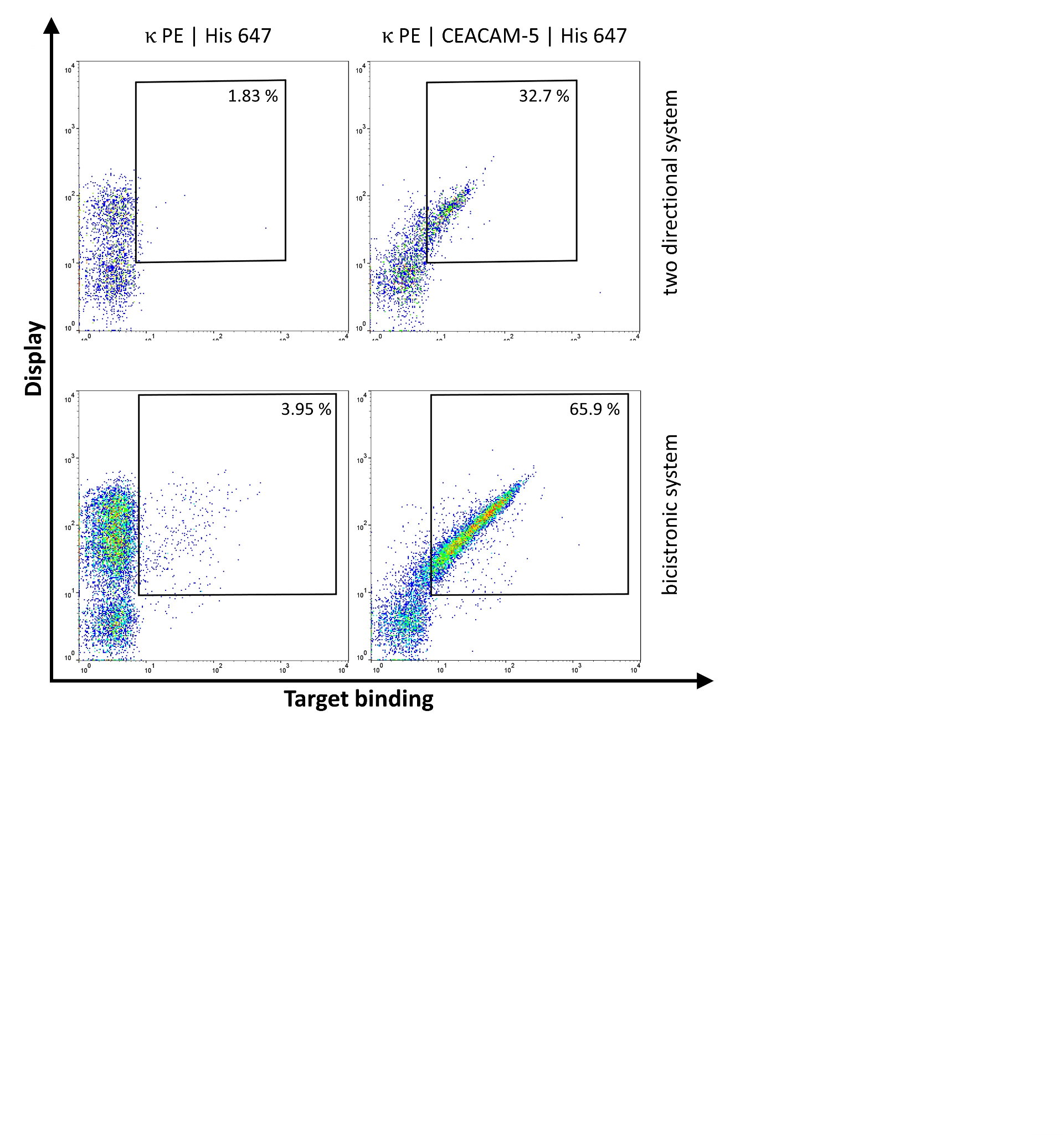


**Figure S3:** Target binding analysis of CEACAM5 immune libraries after three rounds of sorting monitored by flow cytometry. Cells of the two directional (top) and bicistronic (bottom) system were either stained with PE conjugated anti-kappa-antibody, anti-HIS-647 antibody and CEACAM5 (right) or with detection antibodies only (left).


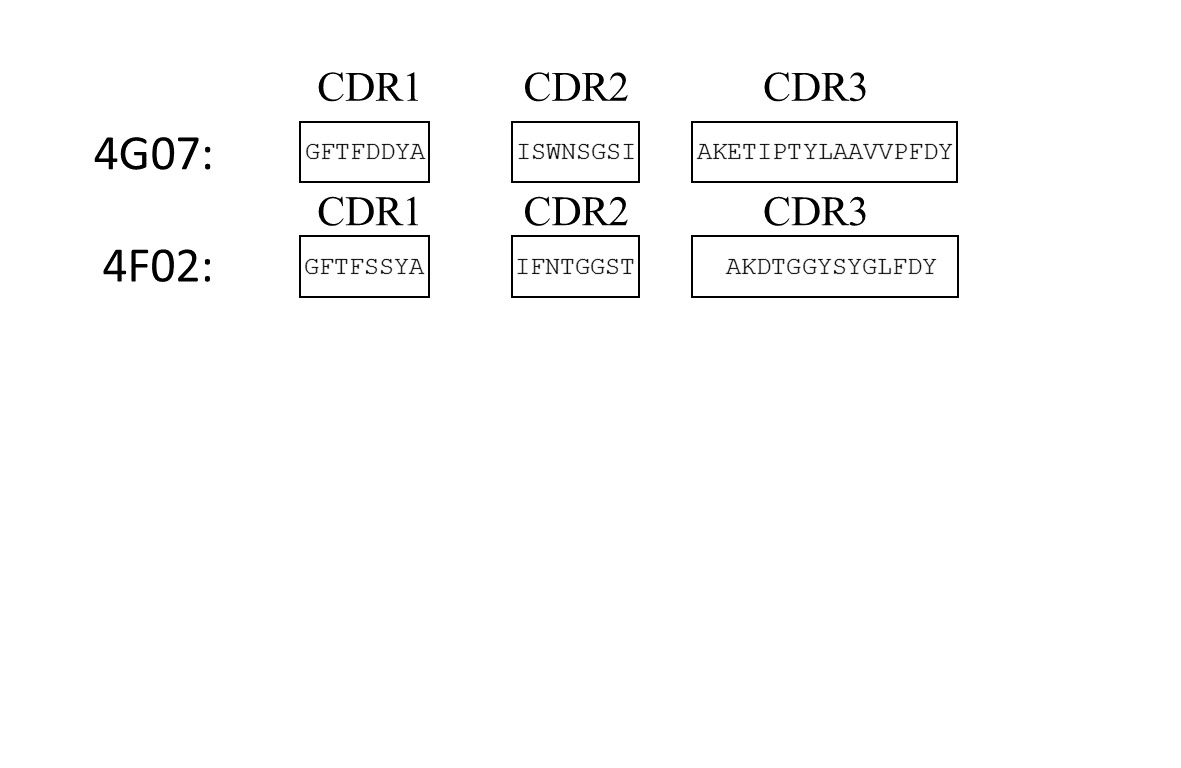


**Figure S4:** Variable heavy chain CDR1-3 sequences of CEACAM5 binding antibodies. 4G07 and 4F02 were the most abundant clones in both libraries after library sorting with approx. 71% (2dir) and 62% (bicis) of sequences clones, and 10% (2dir) and 35% (bicis), respectively.


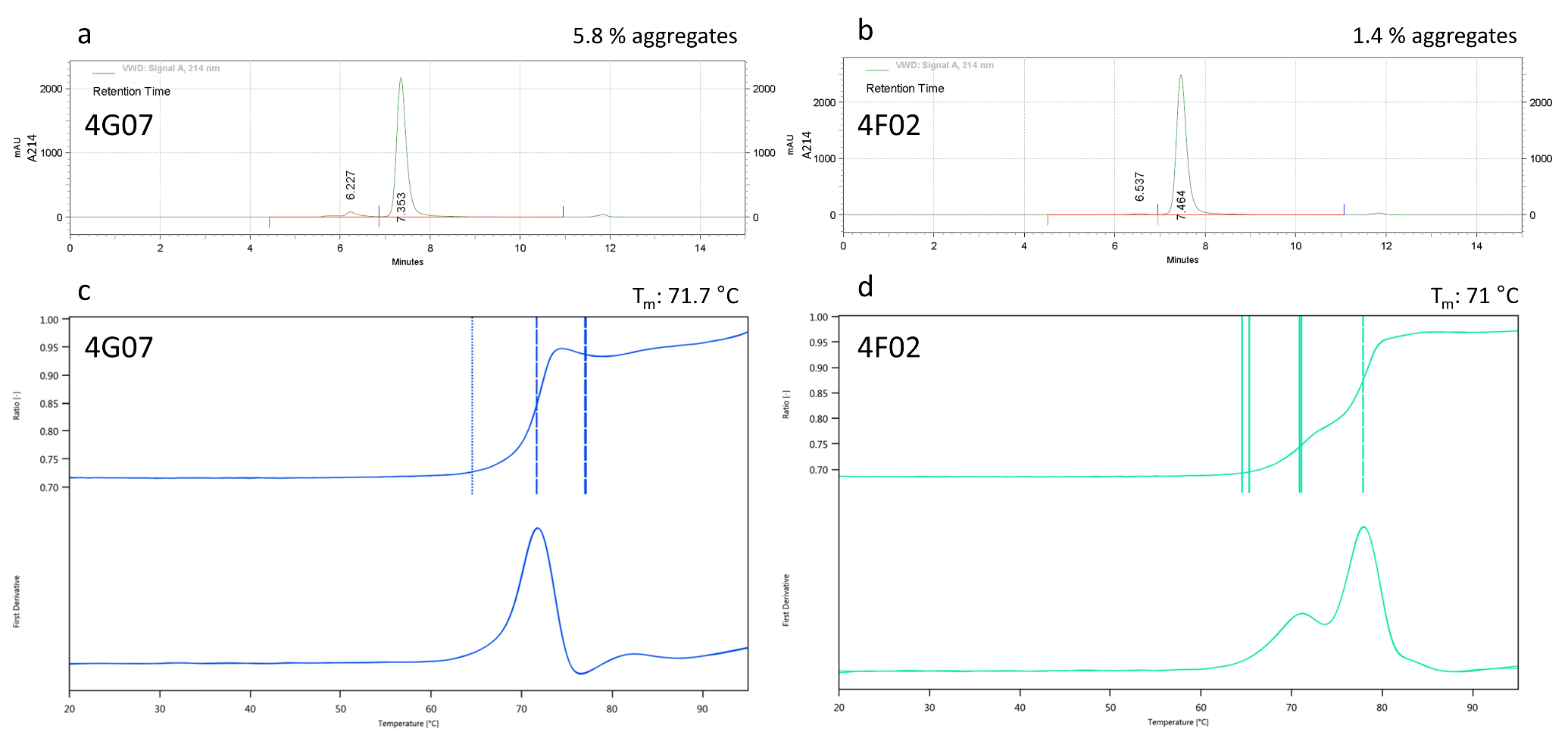


**Figure S5:** Size exclusion chromatography (SEC; a,b with indicated retention times) and differential scanning fluorimetry (DSF; c,d) of variants 4G07 and 4F02. SEC was applied to determine the content of aggregates, monomers and fragments whereas DSF was performed to investigate the characteristic melting temperature of selected antibodies.
